# Effect of Gelation pH on the Entrapment and *In Vitro* Gastrointestinal Digestion of Plant Proteins in Alginate Beads

**DOI:** 10.3390/gels12080717

**Published:** 2026-08-13

**Authors:** Juan Cumilaf, Ever Hernández-Olivas, André Brodkorb, Mónica Rubilar

**Affiliations:** 1 Doctorate in Sciences Engineering with Specialization in Bioprocesses, Faculty of Engineering and Science, Universidad de La Frontera, Temuco 4811230, Chile; 2 Moorepark Research Centre, Teagasc, Fermoy, Co., P61 C996 Cork, Ireland; everhdzo@gmail.com (E.H.-O.); andre.brodkorb@teagasc.ie (A.B.); 3 Department of Chemical Engineering, Faculty of Engineering and Science, Universidad de La Frontera, Temuco 4811230, Chile

**Keywords:** alginate beads, plant proteins, gelation pH, in vitro digestion, colloidal delivery systems, plant-based gel matrices, sustainable functional foods

## Abstract

The development of plant protein-based delivery systems is often limited by poor stability and low retention efficiency under gastrointestinal conditions. This study investigated how pH (4 and 7) during external gelation influences the physicochemical properties, entrapment efficiency (EE), and in vitro gastrointestinal behaviour of alginate beads loaded with hemp protein concentrate (HPC), pea protein concentrate (PPC), or soy protein isolate (SPI). Zeta potential and Fourier transform infrared (FTIR) analyses suggested that at pH 4, the charge profiles of plant proteins favoured electrostatic association with anionic alginate, which was associated with higher EE values, with HPC achieving the maximum of 89.5% at pH 4. Conversely, at pH 7, electrostatic repulsion between biopolymers was associated with reduced EE. During in vitro digestion, beads formulated at pH 4 exhibited greater protein release than those prepared at pH 7, consistent with the expansion of the polymeric network under intestinal conditions. Size exclusion chromatography showed that released proteins underwent extensive proteolysis, generating low-molecular-weight fractions smaller than 300 Da. These results indicate that gelation pH is a relevant processing parameter for modulating protein retention and the extent of protein release of simulated digestion in alginate-based systems, with potential applications in protein-enriched food formulations.

## 1. Introduction

Sodium alginate (SA) is a polysaccharide composed of two linked anionic monomers: β-d-mannuronic acid (M) and α-l-guluronic acid (G) residues, and is mostly obtained from brown seaweed. Due to its biodegradable, biocompatible, and non-toxic properties, SA has attracted attention for its applications in food science and bioencapsulation technologies [1,2,3,4,5,6]. Alginate can be cross-linked through external or internal gelation using polyvalent cations such as Ba^2+^, Ca^2+^, and Sr^+2^; this means they can physically form a gel in the presence of these ions [3,4,5,6]. The external gelation method involves gel formation through the diffusion of ions into an alginate solution, creating an egg-box-like structure between the carboxyl group of the alginate and the polyvalent cation [7]. For this process, drops of alginate solution are typically extruded through a syringe nozzle into a calcium salt bath, which is commonly known as conventional or classic spherification.

Recent studies have investigated the gastrointestinal release behaviour of encapsulated compounds from alginate-based beads [8,9,10,11,12,13,14]. Due to the pH-dependent ionization of alginate carboxyl groups, these hydrogel matrices undergo distinct structural changes, including swelling, network relaxation, and matrix disintegration, under different gastrointestinal conditions [12,14]. In the acidic gastric environment, protonation of carboxyl groups reduces electrostatic repulsion within the network, which can limit swelling and delay the release of encapsulated compounds [9,11,12,15]. Upon transition to the neutral or slightly alkaline intestinal environment, deprotonation of carboxyl groups promotes electrostatic repulsion and network swelling, while ion exchange with physiological cations may contribute to progressive network relaxation and compound release [8,10,13]. Consequently, the recent literature highlights that the physicochemical properties of the alginate matrix influence swelling behaviour, network relaxation, and the release of encapsulated compounds during simulated digestion [8,11,13,15], providing a foundation for the development of composite systems incorporating additional biopolymers, such as proteins, to further modulate their digestive performance.

Although alginate systems have been extensively studied as wall material for the encapsulation systems, the role of protein–polysaccharide interactions in matrix integrity and gastro-intestinal behaviour are not yet fully understood. In this regard, the use of proteins as co-structuring agents has been a common strategy, including whey protein isolate [16,17,18]. However, interest in replacing it with plant-based alternatives has been growing, driven by global population growth, the environmental impact of animal protein production, increased interest in plant-based diets, the valorisation of agro-industrial waste, and the need to offer more options to consumers [19,20,21]. Among the most promising plant proteins, hemp protein, pea protein and soy protein have attracted growing interest due to their composition, functional properties, and relevance in the development of plant-based foods [22,23]. Soy protein isolate (SPI), pea protein concentrate (PPC), and hemp protein concentrate (HPC) were selected for this study as they represent three distinct categories of plant-based proteins: soy as an established industry benchmark, pea as a rapidly growing mainstream alternative valued for its hypoallergenic profile, and hemp as an emerging, sustainable source often derived from agro-industrial byproducts [24,25,26,27]. Furthermore, these proteins exhibit different isoelectric points (pI) and solubility profiles. Evaluating them allows for a comprehensive assessment of how varying protein charge characteristics influence electrostatic interactions with negatively charged alginate during external gelation at acidic (pH 4) versus neutral (pH 7) conditions. Bead stability during gastrointestinal transit determines the release of entrapped compounds [8,28]. Therefore, in vitro digestion models are essential to evaluate how pH-dependent alginate–protein interactions influence protein retention under simulated gastric and intestinal conditions. Additionally, the intrinsic buffering capacity of plant proteins modulates this process by attenuating local pH gradients at the bead interface during gastric acidification [29,30].

However, a specific knowledge gap remains regarding how the gelation pH, relative to the isoelectric point (pI) of each plant protein, influences electrostatic interactions during network formation and, consequently, the structural integrity and protein retention of the beads during gastrointestinal digestion. Although previous studies have investigated plant protein–alginate systems, few have systematically evaluated how the choice of gelation pH (acidic versus neutral) affects entrapment efficiency and protein release behaviour across proteins with distinct isoelectric points.

Thus, the pH determines the net charge of plant proteins according to their isoelectric point (pI), conditioning their interaction with the anionic polysaccharide alginate during gelation [31]. At pH 4, SPI (pI~4.5) and PPC (pI~4.7) exhibit near-zero net charge, whereas HPC (pI~5) retains a positive charge and favours electrostatic attraction with anionic alginate; in contrast, at pH 7, all proteins acquire a negative charge (pH > pI), generating repulsion. These charge-dependent interactions influence network organization, affecting its stability and protein release, thereby indirectly influencing protein hydrolysis during simulated digestion. The aim of this study was to study how the pH (4 and 7) during external gelation modulates the electrostatic interactions between alginate and plant proteins (HPC, SPI, and PPC), thereby affecting matrix organization and, ultimately, controlling protein entrapment and stability during simulated gastrointestinal digestion. Bead size and sphericity were evaluated as structural indicators of matrix organization, while protein retention capacity and the extent of proteolytic hydrolysis were used to assess digestive stability.

## 2. Results and Discussion

### 2.1. Plant-Based Protein Solubility

Protein solubility is a key functional and thermodynamic parameter that influences technological properties such as emulsification and gelation. It can be defined as the maximum amount of protein that dissolves in a solvent under specific conditions and is determined by both environmental factors (pH, temperature, and pressure) and intrinsic characteristics such as molecular weight, polarity, and amino acid composition [32,33,34].

**Figure 1:**
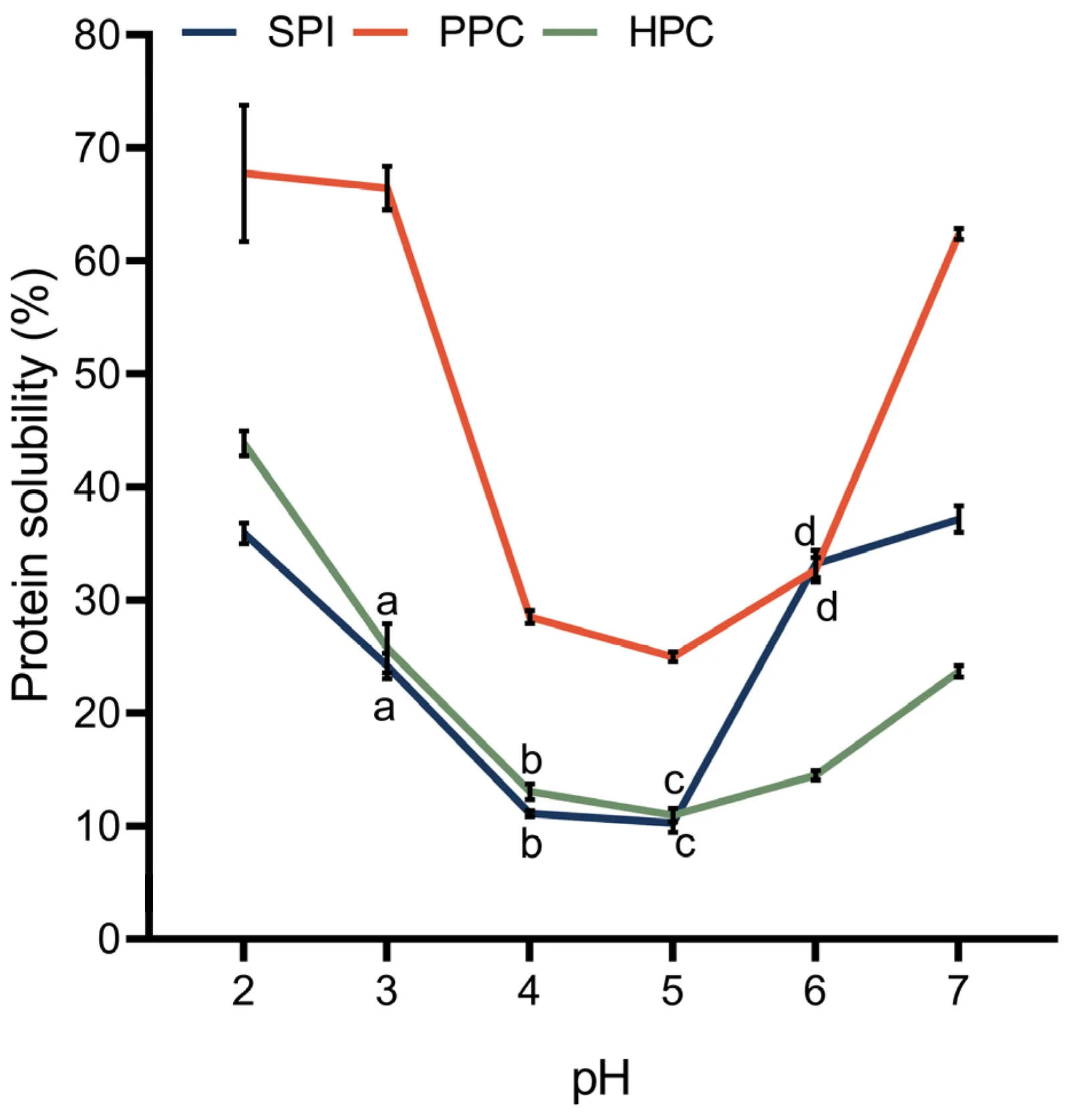
Effect of pH on the solubility of PPC, HPC, and SPI (0.1% *w*/*v*). Data represent the mean ± SD of triplicate measurements. Different letters indicate significant differences among means (*p* < 0.05).

The solubility profiles of plant proteins (PPC, HPC, and SPI) at different pH values are shown in Figure 1. In general, PPC showed a significantly higher solubility (*p* < 0.05) than SPI and HPC across the entire pH range (2–7). Among the proteins analysed, solubility peaked at pH 2, with values of 67.7%, 43.8% and 35.9% for PPC, HPC and SPI, respectively. Overall, the results showed that pea protein concentrate has a greater ability to solubilise in water across the entire range evaluated compared to soy and hemp proteins, which is not surprising given that PPC was purified by removal of most insoluble protein. Most commercially available PPCs exhibit poor to average water solubility, which renders them not usable as core material for gel encapsulation.

As expected, all proteins exhibited a pH-dependent solubility pattern, with minimum values observed near their isoelectric points (pI). At the pI, the net surface charge approaches zero, reducing electrostatic repulsion and favouring aggregation, which leads to a decrease in solubility. When the pH deviates from the pI, the functional groups of the proteins are protonated or deprotonated, which modifies the overall net charge and increases electrostatic repulsion. This behaviour explains the higher solubility observed under acidic conditions [35,36]. The solubility of each protein was measured separately to establish a baseline behaviour before studying the interactions between the proteins and alginate. Although measuring the solubility of the protein–alginate complexes would provide additional information, the solubility profiles of each protein allow us to understand the intrinsic behaviour of each one and to interpret subsequent interactions with alginate. A finer pH gradient (e.g., 0.5-unit intervals) around the isoelectric point could provide a more detailed characterization of solubility patterns within the pI region (4.5–6.0). However, the pH values selected for gelation (4.0 and 7.0) represent conditions that are clearly below and above the pI range, allowing us to compare different electrostatic scenarios.

### 2.2. Zeta Potential

Zeta potential measurements were performed to characterise the apparent surface charge of individual proteins (SPI, HPC, PPC) and sodium alginate under varying pH conditions. While measuring the zeta potential of protein–alginate complexes would provide direct evidence of complex formation, the individual charge profiles allow us to predict and interpret the electrostatic interactions between components. The charge state of each component at different pH values determines whether attractive or repulsive forces will dominate during gelation, which is critical for understanding the observed differences in entrapment efficiency and bead properties.

**Figure 2:**
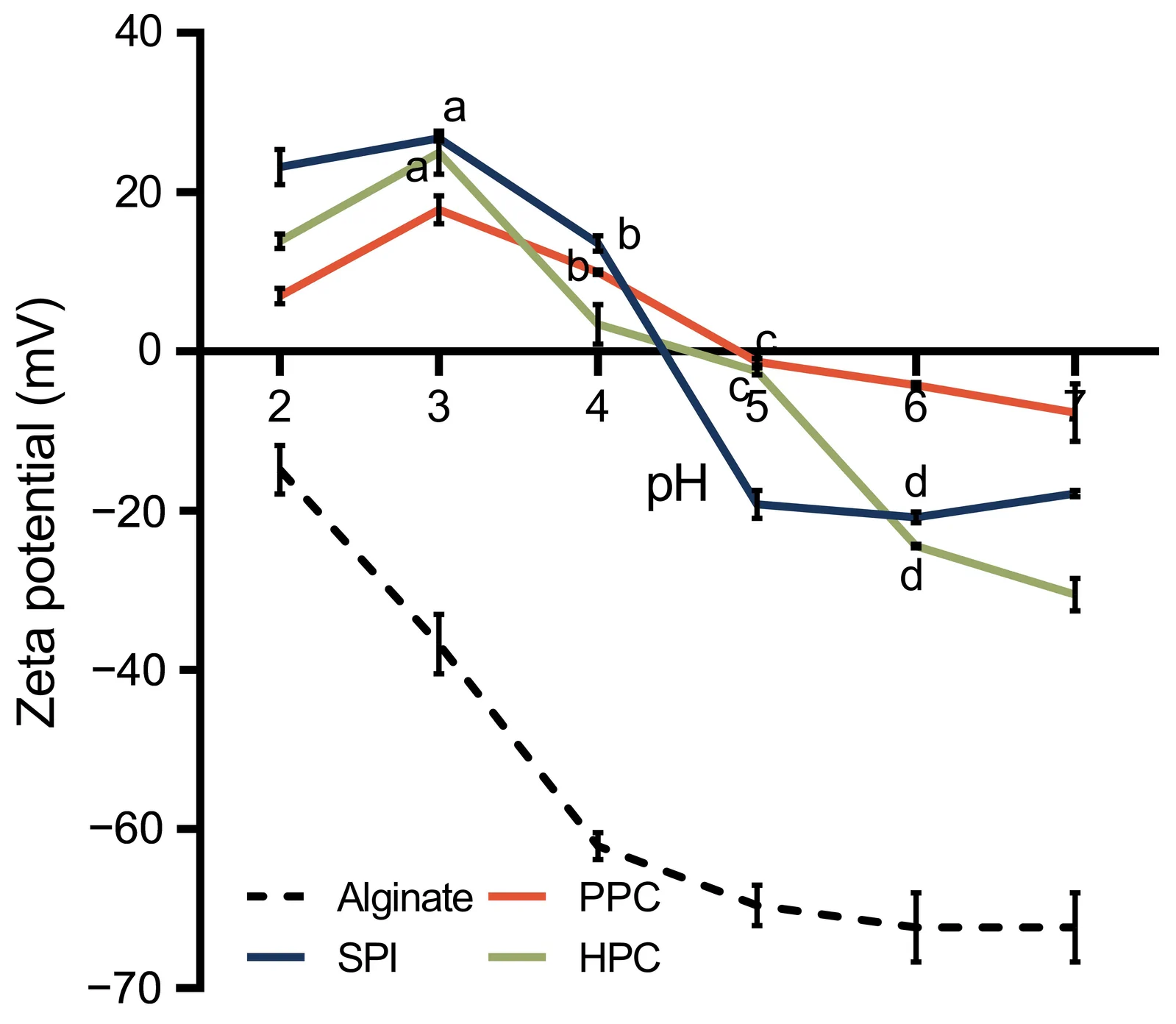
Zeta potential as a function of pH for 0.1% (*w*/*v*) SPI, PPC, HPC, and alginate in water. Data represent the mean ± SD of triplicate measurements. Means sharing the same letter are not significantly different (*p* < 0.05).

The zeta potential values of SPI, HPC, PPC and alginate at different pH values are shown in Figure 2. All proteins exhibited similar pH-dependent behaviour: their surface charge was positive at acidic pH (below their pI) and became negative above the pI. In contrast, alginate exhibited a consistently negative zeta potential across the entire pH range evaluated. At pH 2, all plant proteins showed positive charges, with a surface charge of approximately +35 mV (PPC), +28 mV (SPI) and +25 mV (HPC). The charge decreased progressively as the pH increased, reaching a net charge of zero around pH 4–5 and becoming negative under neutral conditions (between −30 and −24 mV at pH 7). This is attributed to the protonation–deprotonation of ionisable functional groups and explains the reduced solubility observed near the isoelectric point. Alginate remained negatively charged throughout the entire pH range, showing no significant variations (*p* > 0.05) for pH ≥ 5, which is typical of its anionic nature.

The zeta potential represents the apparent surface charge of particles in an aqueous medium, which directly influences the stability of colloids in suspension. A high absolute value of the net charge indicates greater electrostatic repulsion between particles and generally a more stable dispersion. Values close to zero favour aggregation and sedimentation, generally leading to unstable and less soluble suspensions [36,37,38,39].

Since sodium alginate remained negatively charged across the entire pH range, the electrostatic attraction or repulsion between alginate and proteins depends on their respective charge states. At pH values below the protein pI, proteins are positively charged and can interact electrostatically with alginate, which could reduce the availability of Ca^2+^ binding sites during ionic gelation. This may result in weaker and more porous gels. Conversely, when both components have negative charges, electrostatic repulsion may limit their association, decreasing protein retention within the gel network [15,40,41]. These interactions can influence not only the microstructure of the gel but also its functional performance, including the efficiency of protein trapping and release during gastrointestinal digestion.

### 2.3. Interactions Between Plant-Based Proteins and Alginate

Building on the solubility and surface charge characteristics discussed in Section 2.1 and Section 2.2, the structural behaviour of plant proteins and alginate in aqueous systems was further investigated using FTIR spectroscopy. Protein solubility varied with pH, consistent with the apparent surface charge profiles that reflect the protonation–deprotonation behaviour of ionisable groups [42,43]. To obtain molecular insight into how these charge-dependent interactions influence protein conformation, FTIR spectroscopy was used to characterize the change in secondary structure of PPC, HPC, and SPI, in their isolate state and when combined with alginate at pH 4 and 7. These pH values were selected based on the charge profiles discussed in Section 2.2. Specifically, pH 4 represents conditions that generally favour protein–alginate association, although the dominant interaction mechanism differs depending on each protein’s proximity to its pI: electrostatic attraction predominates for HPC, which retains a net positive charge, whereas hydrogen bonding, hydrophobic interactions, and localized positive charge patches become more relevant for SPI and PPC, whose net charge approaches zero near their respective pI. Conversely, pH 7 represents repulsive conditions where both biopolymers carry a net negative charge.

**Figure 3:**
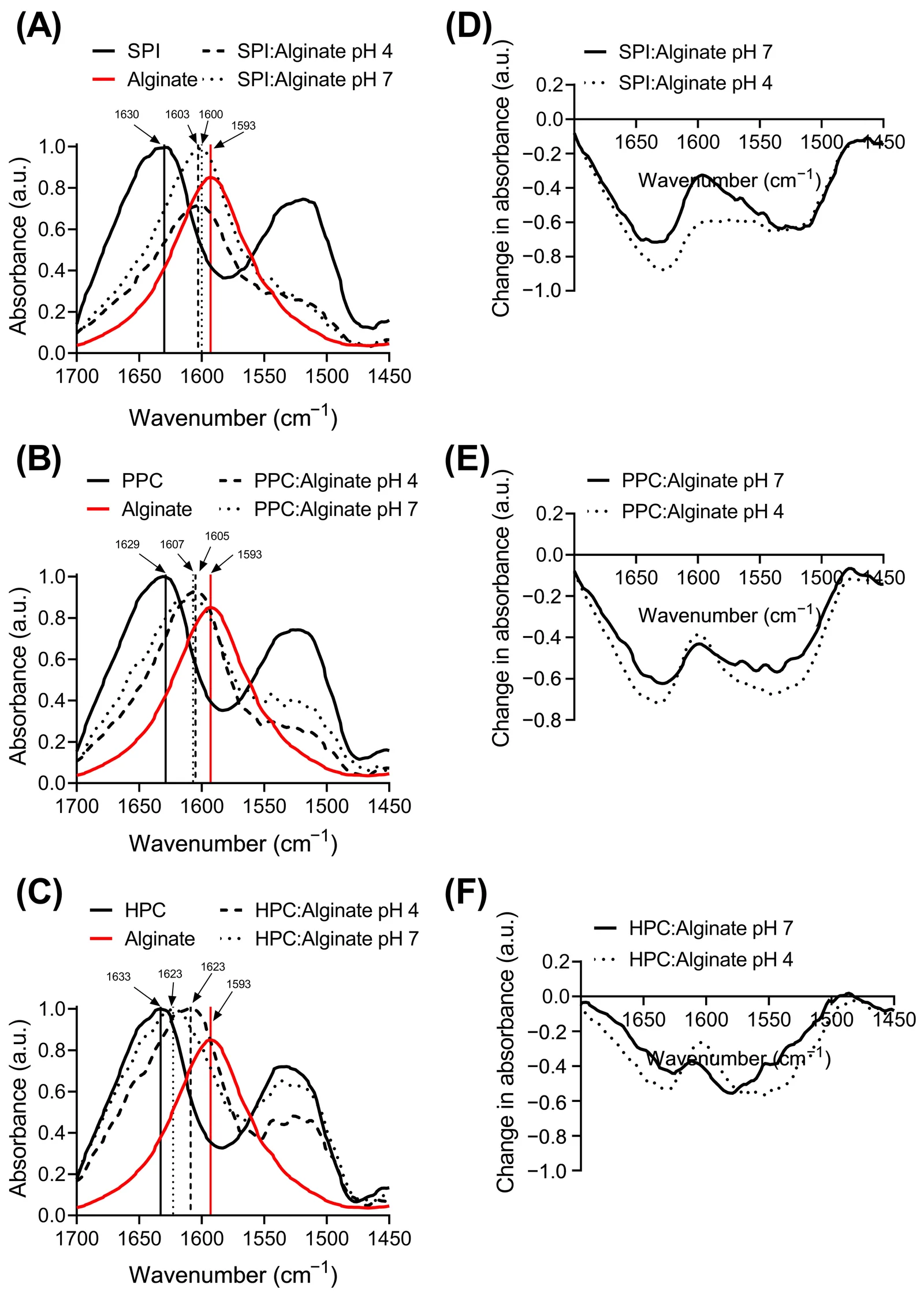
FTIR spectra of pure proteins, alginate, and protein–alginate complexes prepared at pH 4 and pH 7 (**A**–**C**), and the corresponding difference spectra obtained by subtracting the spectra of the pure components from the complexes (**D**–**F**) in amide I and II.

Figure 3 (panels A–C) shows variations in the amide I and II regions. These spectral changes may reflect conformational rearrangements influenced by pH and the presence of alginate, where molecular interactions may take place via the formation of alginate–protein complexes. For HPC, attractive electrostatic forces are the primary driving force, whereas for SPI and PPC, which are near their pI at pH 4, complexation is primarily facilitated by non-covalent interactions and charge heterogeneity on the protein surface [9,44]. As shown in Figure 3A–C, the incorporation of alginate at both pH 4 and pH 7 caused changes in the amide I and II bands, while the subtracted spectra (Figure 3D–F) highlight the specific wave number regions most affected by these interactions. The amide I peak associated with the β-sheet structures of all proteins (1629–1633 cm^−1^) shifted to lower wave numbers (1600–1623 cm^−1^) after pH variation and alginate addition [45,46].

These downward shifts are typically associated with stronger intermolecular interactions and reduced molecular mobility, consistent with the formation of more ordered β-sheet-type protein–polysaccharide assemblies or intermolecular β-sheet between proteins [47,48]. In the case of PPC, the amide I peak shifted from 1629 cm^−1^ to 1605 cm^−1^ (pH 4) and 1607 cm^−1^ (pH 7). HPC showed shifts from 1633 cm^−1^ to 1609 and 1623 cm^−1^ at pH 4 and 7, respectively. In the SPI and alginate mixtures, the amide I peak shifted from 1630 cm^−1^ to 1603 cm^−1^ (pH 4) and 1600 cm^−1^ (pH 7).

The amide II peaks, located between 1470 and 1570 cm^−1^, correspond mainly to C-N stretching and N-H bending vibrations [49]. In native proteins, these peaks appear between 1519 and 1527 cm^−1^, but their intensity decreases in the presence of alginate, indicating conformational reorganisation and the formation of hydrogen bonds that immobilise parts of the protein chain [48,49,50].

Of the three proteins, PPC showed the most pronounced changes in the amide I region (Figure 3B,E), suggesting that PPC may undergo more pronounced conformational changes upon alginate binding [51]. HPC showed moderate spectral variations (Figure 3C,F), while SPI (Figure 3A,D) showed more pronounced decreases in amide II intensity, consistent with hydrogen bond formation and extensive conformational rearrangement [52,53]. The more pronounced negative bands observed at pH 4 in the differential spectra are consistent with protein–alginate association. For HPC, this involves electrostatic attraction between protonated amino acid residues and alginate carboxylate groups [54,55]. For SPI and PPC, despite their near-zero net charge, association is governed by multiple intermolecular forces, including hydrogen bonding, hydrophobic contributions from exposed non-polar residues, and binding to localized positive charge patches [54,55,56]. These intermolecular forces may promote conformational rearrangements and protein aggregation near their respective isoelectric points, which are reflected in the observed shifts in the amide I and II bands [45].

Subtracting the absorbance of the individual components allowed the effects of pH and biopolymer interactions to be isolated. Spectral changes in the amide I and II regions were more pronounced for alginate–protein mixtures at pH 4, indicating stronger overall intermolecular interactions, electrostatic for HPC, and non-covalent for SPI and PPC, and more extensive conformational rearrangements under acidic conditions [57,58].

### 2.4. Alginate–Protein Bead Structure

**Figure 4:**
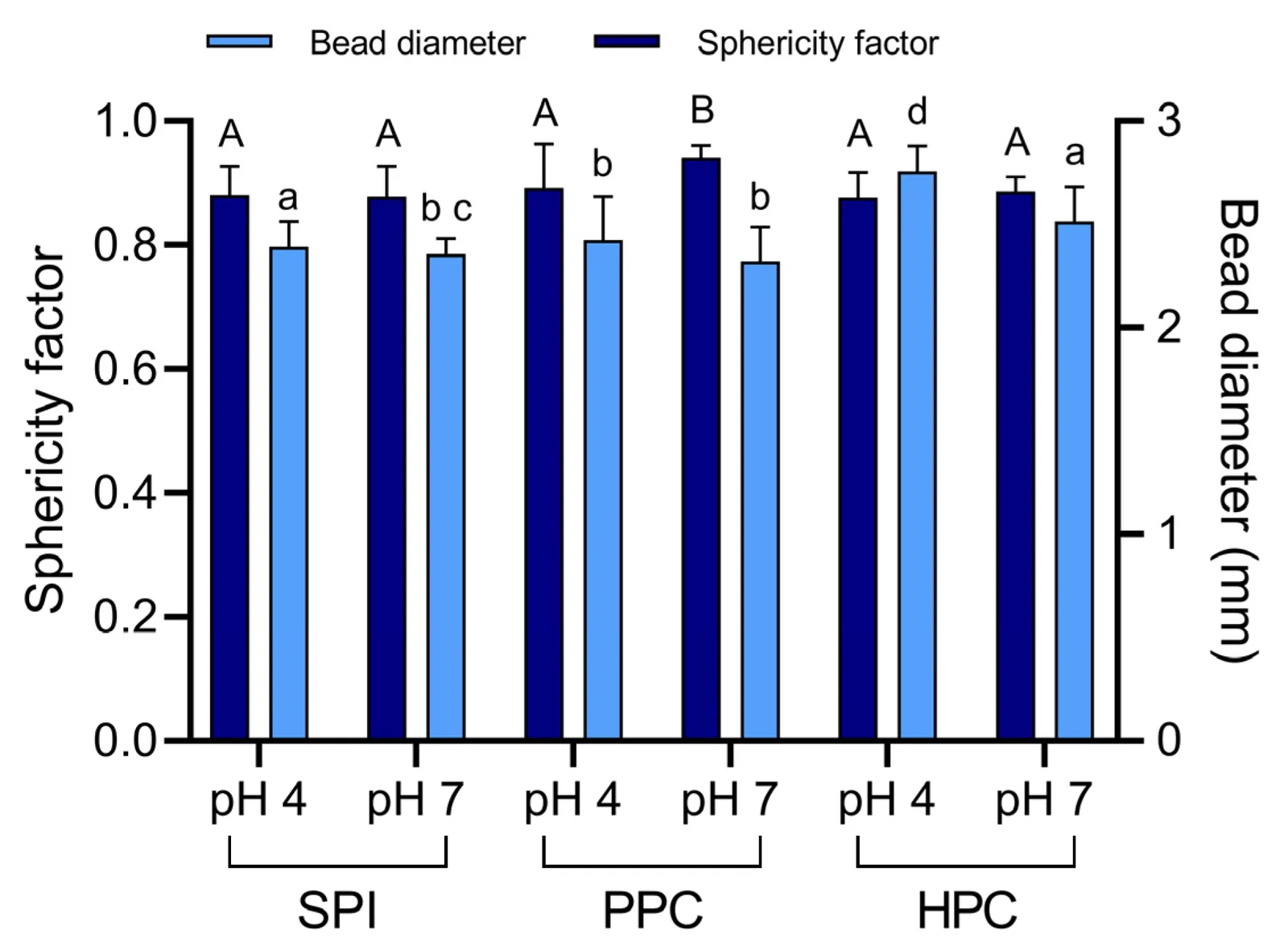
Sphericity factor and bead diameter of alginate beads loaded with SPI, PPC, and HPC at pH 4 and 7. Data represent means ± SD (*n* ≥ 60). Bars not sharing the same letter are significantly different (*p* < 0.05).

Mean structure diameter and sphericity factor were measured for alginate beads prepared with each protein at different pH conditions, results are shown in Figure 4. The sphericity factor is a dimensionless value that describes the extent to which the shape of an object approximates that of a perfect sphere. It is generally used to evaluate the shape of structures and their characteristics. SF equal to 1 implies a perfect sphere shape; however, according to Chan et al. [59], beads whose SF value is greater than or equal to 0.905, will still be considered as spheres.

**Figure 5:**
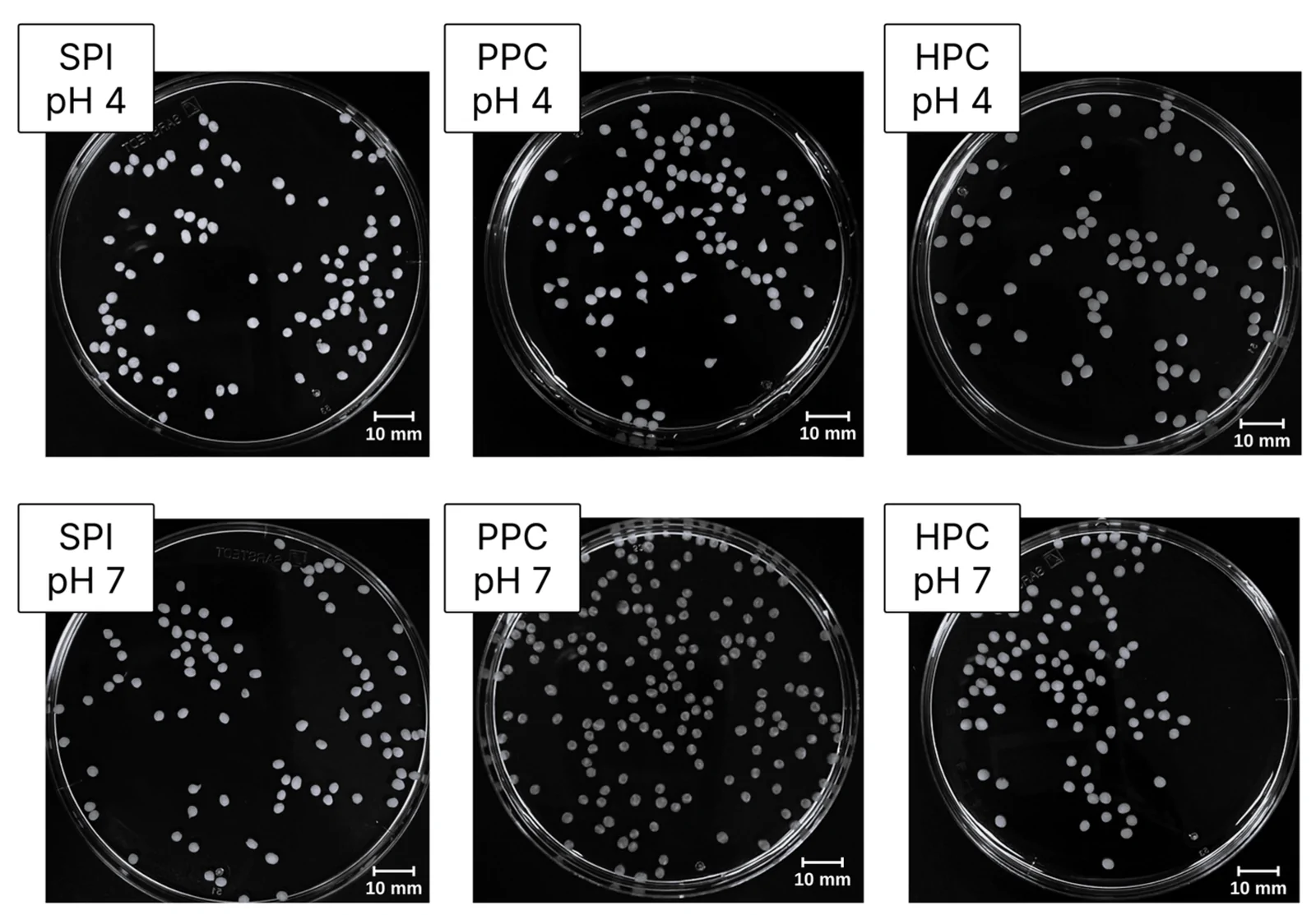
Macroscopic appearance of alginate beads loaded with SPI, PPC, and HPC prepared by ionic gelation at pH 4 and pH 7. Scale bars = 10 mm.

The sphericity factor of the hydrogel beads produced at pH 4 were 0.89, 0.88 and 0.87 for PPC, SPI, and HPC, respectively. On the other hand, at pH 7, this factor reached values of 0.94 for PPC, 0.88 for SPI and 0.89 for HPC; therefore, in most cases the alginate–protein beads cannot be considered spherical particles and were classified as semispherical beads, as can be observed in Figure 5. According to Lee et al. [60] the shape of the alginate–protein beads and their representation through the sphericity factor (SF) can influence their mechanical and chemical stability, has been reported higher strength for gels whose SF was closer to 1. Since spherical beads tend to have a better surface area/volume ratio, improving their resistance to breakage and therefore the release of encapsulated materials.

Beads formulated at different pHs have shown different sizes and their mean diameters were between 2.32 and 2.76 mm, PPC–alginate beads formed at pH 7 were the smaller particles compared to HPC–alginate at pH 4 which were the largest ones. In general, all the beads produced showed a smaller diameter when formulated at pH 7. Although the alginate-PPC beads at pH 7 are statistically more spherical than the rest of the structures obtained with other proteins and pH values, it is not possible to conclude that the formulation of beads at pH 7 allows obtaining more spherical structures compared to those produced at pH 4. On the other hand, the mean size of the bead structures has been significantly smaller when the formulation pH was 7 for hemp and soy proteins. In addition, although the values did not reach statistical significance, the general trend suggests a consistent effect. This can be explained by the pH-dependent behaviour of alginate: at acidic pH, protonation of carboxyl groups restricts the formation of ionic bridges between alginate chains and Ca^2+^, resulting in a less densely crosslinked network [61,62].

### 2.5. Protein Entrapment Efficiency

**Figure 6:**
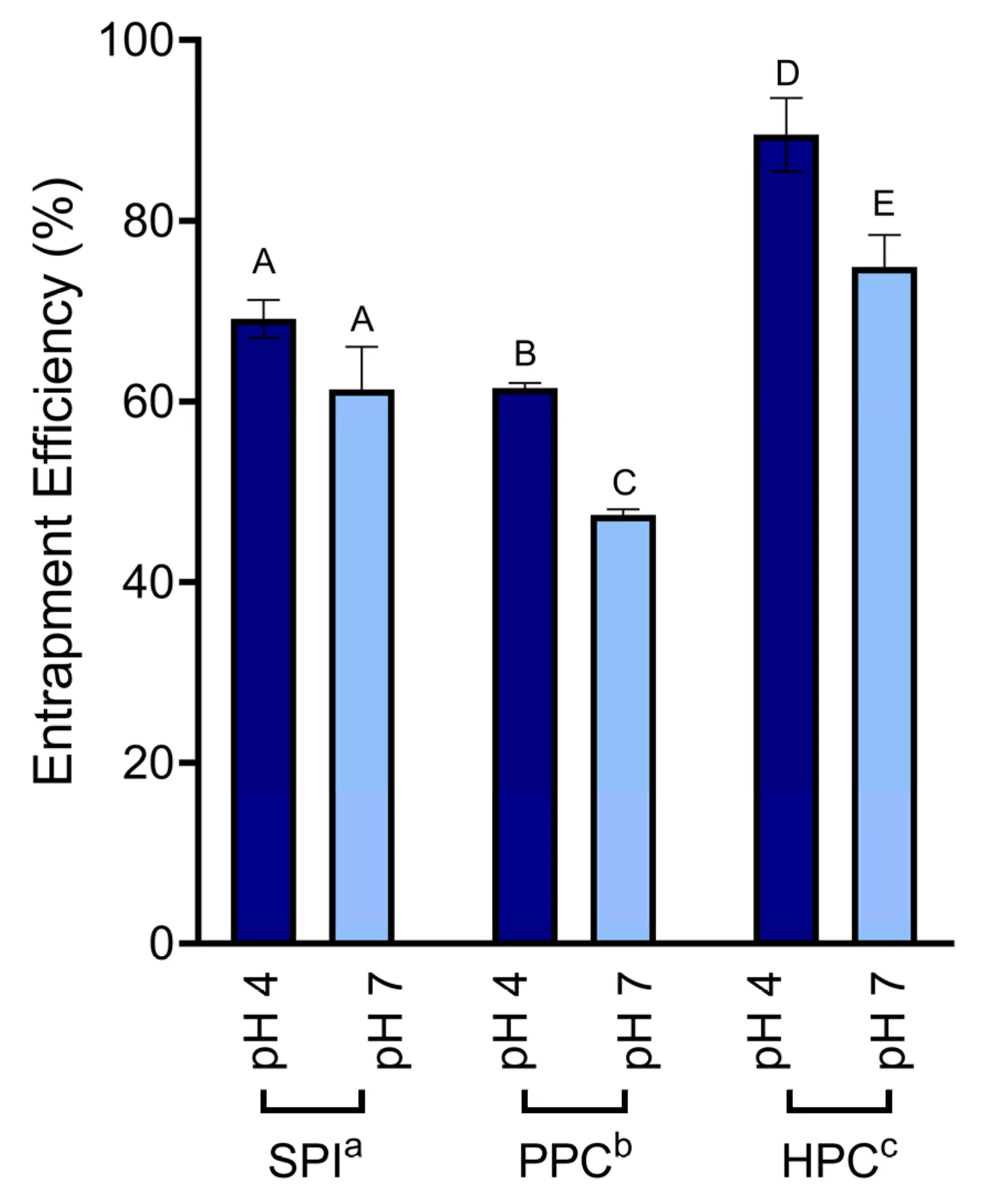
Effect of pH (4 and 7) on protein entrapment efficiency in alginate beads. Different letters present statistically significant differences (*p* < 0.05). Uppercase letters indicate comparisons between different pH values (4 and 7) for the same alginate–protein system. Lowercase letters indicate comparisons between different protein types for the same pH value.

Protein Entrapment Efficiency (EE) for the alginate–protein beads is shown in Figure 6. Beads formulated at pH 4 showed a higher capacity to trap protein than beads formulated at pH 7. Among the alginate–protein beads formulated at pH 4, HPC showed the highest entrapment efficiency (89.52%), compared to SPI (69.13%), and PPC (61.49%). Regarding those formulated at pH 7, similar results were observed, with HPC reaching 74.88%, while SPI and PPC reached 61.35% and 47.42%, respectively. The higher entrapment values observed for beads prepared at pH 4 are likely associated with the electrostatic attraction between the positively charged HPC (pI~5) and the anionic alginate, as supported by the zeta potential results [63,64]. However, attributing this behaviour solely to electrostatics is an oversimplification, as protein-polysaccharide complexation is governed by multiple intermolecular interactions [48,65]. At pH 4, SPI and PPC are close to their isoelectric points and therefore exhibit minimal net charge, whereas HPC retains an overall positive charge. At this acidic pH, partial protonation of alginate carboxyl groups (pKa~3.38–3.65) may also promote hydrogen bonding with the amide and amine groups of the proteins [61]. This interpretation is supported by the spectral shifts observed in the amide I and II regions of the FTIR spectra (Section 2.3). Furthermore, the reduced overall electrostatic repulsion near the pI region may allow hydrophobic patches on the hemp protein to interact with the polymer matrix or self-associate [35,48,64]. Together, these interactions provide a plausible explanation for the higher entrapment efficiency of HPC under acidic gelation conditions.

These combined intermolecular interactions facilitate the incorporation of proteins into the alginate network, thereby enhancing the ability of alginate to function as an effective protein carrier [48]. On the other hand, different levels of entrapment were attributed to the intrinsic techno-functional properties of the used protein. In the context of ionic gelation, protein displacement from the alginate bead occurs primarily because its solubility in the medium modulates the diffusion rate through the polysaccharide matrix into the gelling bath. This diffusion process is driven by the concentration gradient, moving the protein from a zone of high concentration (inside the bead) to a zone of low concentration (the gelling bath), directly affecting entrapment efficiency value [66].

**Figure 7:**
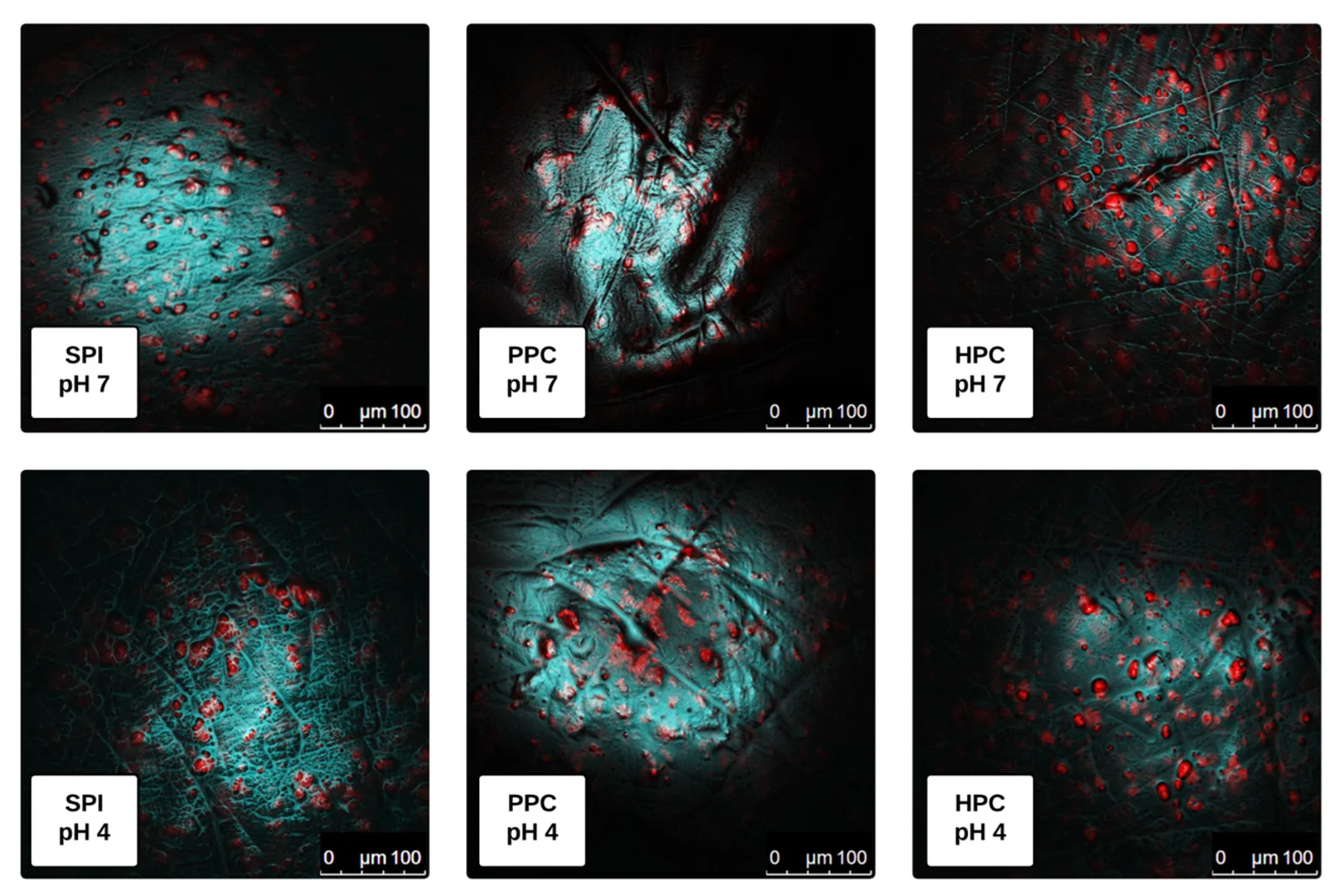
Surface microstructure of alginate–protein beads; spatial distribution of alginate (shown in cyan, labelled with FITC) and plant proteins (shown in red, labelled with Fast Green) in HPC, SPI and PPC beads formulated at pH 4 and pH 7.

Confocal laser scanning microscopy (CLSM) images (Figure 7) revealed the distribution of proteins within the alginate–protein beads and confirmed successful protein loading. The micrographs showed that proteins were distributed throughout the bead matrix and appeared as clustered vesicle-like structures of homogeneous size. Surface images further demonstrated the presence of protein in all samples, indicating effective incorporation of HPC, SPI, and PPC into hydrogels prepared at both pH 4 and pH 7.

### 2.6. Behaviour of Alginate–Protein Beads During In Vitro Gastrointestinal Digestion

#### 2.6.1. Protein Release

**Figure 8:**
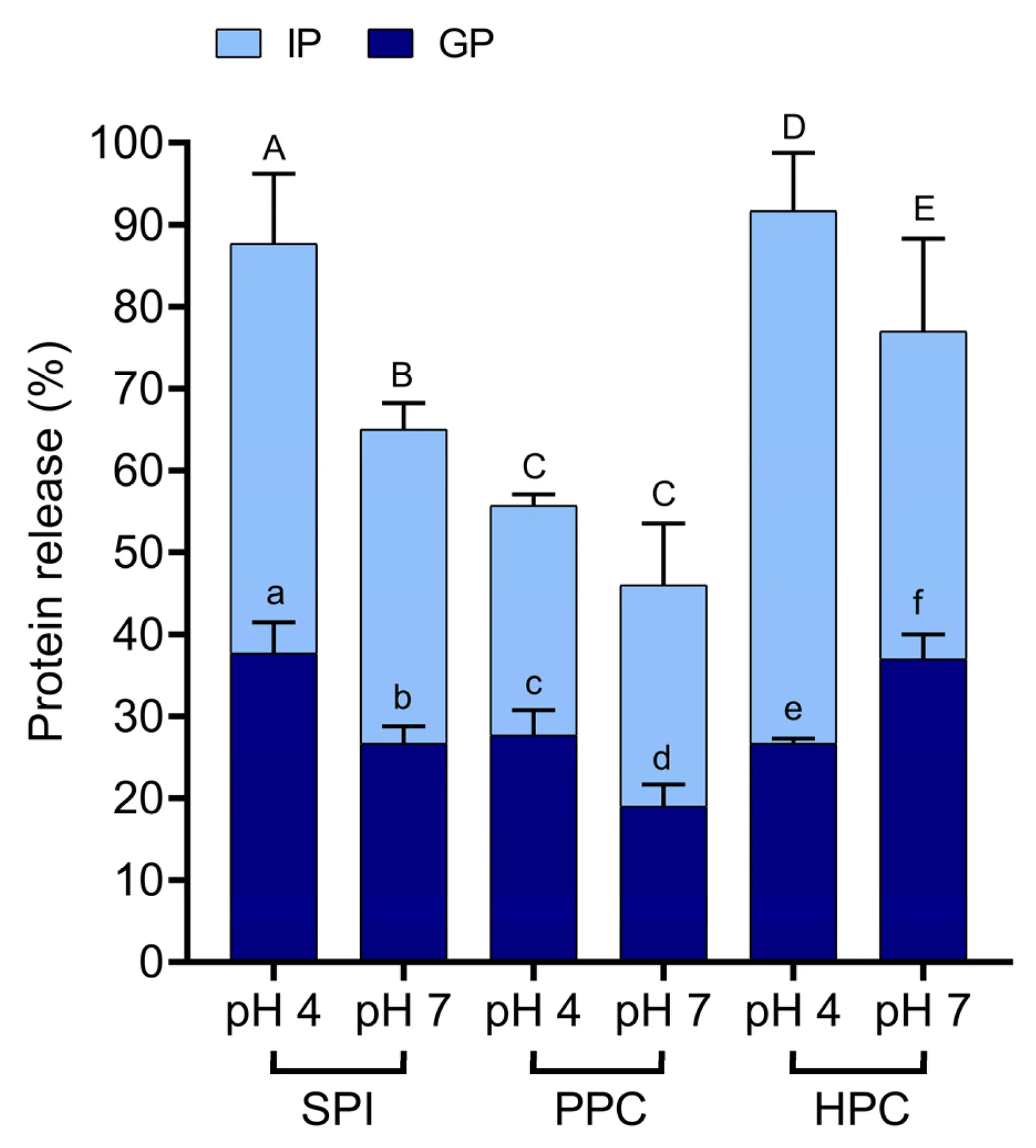
Protein release for each alginate–protein bead at the end of the gastric phase (GP) and intestinal phase (IP). Values with different letters indicate statistically significant differences (*p* < 0.05). Uppercase letters denote comparisons between beads produced at pH 4 and 7 for the same alginate–protein system during the intestinal phase. Lowercase letters denote comparisons between beads produced at pH 4 and 7 for the same alginate–protein system during the gastric phase.

Protein release from each alginate–protein structure produced by the external gelation method is presented in Figure 8. During the static gastric stage (GS) at pH3, protein release from beads formulated at pH 4 ranged from 19.0% (ALG–PPC) to 37.7% (ALG–SPI). Moreover, at pH 4, HPC– and PPC–alginate beads released significantly lower amounts of protein than the SPI–alginate beads (*p* < 0.05). For beads formulated at pH 4, no significant differences were observed between HPC–alginate and PPC–alginate beads (*p* > 0.05). At the end of the intestinal digestion, the release of protein encapsulated by alginate has shown differences over the type of protein and pHs used. Beads produced at pH 7 were able to release less amount protein than ones made under acidic pH value. Thus, at pH 4 SPI and HPC alginate beads were not significant different between them during intestinal stage. However, both showed significantly higher protein release (*p* < 0.05) than the PPC–alginate beads. Any type of alginate–protein beads produced at pH 7 showed no significant difference for protein releasing (Tukey’s test considered *p* < 0.05).

When the environmental pH is lower than the beads, the hydrogels’ molecular interactions formed during the external gelation and other chemical interactions during the processing (e.g., carboxyl groups protonated during alginate pH adjusting or protein net charge) will tend to remain stable. On the other hand, during the simulated intestinal stage, the increase in pH ionizes the carboxyl groups of alginate. This pH-responsive swelling behaviour is characteristic of alginate-based hydrogel networks, where the transition from protonated carboxyl groups, which favour hydrogen bonding and network contraction in acidic environments, to deprotonated carboxylate anions increases the matrix pore size and facilitates the release of entrapped compounds [12]. This phenomenon occurs mainly in beads formulated at pH 4. On the contrary, in beads produced at pH 7, protein release may be governed by other mechanisms, such as mass transfer processes. These processes depend largely on structural parameters, including porosity (the fraction of void space) and tortuosity (a measure of the convolutedness of diffusion pathways within the gel matrix). Furthermore, the mesh size and crosslinking density of the polymeric network act as selective physical barriers that restrict the diffusion and mass transfer of the entrapped proteins [14]. Higher tortuosity implies longer more winding paths for protein diffusion, which can reduce the extent of protein release [67,68]. Furthermore, the lower protein release observed in the gastric fluid for beads formulated at pH 7 can be mechanistically explained by their structural and electrostatic properties. As demonstrated by Chuang et al. [61], more spherical alginate particles inherently possess a lower surface-area-to-volume ratio compared to aspheric or oblate particles formed under acidic conditions, which physically restricts the initial mass transfer and diffusion rate of the entrapped compound. Additionally, at pH 7, the electrostatic repulsion between the negatively charged alginate matrix and the negatively charged plant proteins limits the formation of a highly porous, open network, promoting a more compact and homogeneous microstructure with increased internal tortuosity [69]. When combined with the protonation of alginate carboxyl groups in the acidic gastric environment [61], this relatively more spherical and denser matrix remains relatively intact, severely restricting gastric fluid penetration and protein diffusion. While direct microstructural characterization was beyond the scope of this study, the observed release behaviour is consistent with previous reports indicating that protein–alginate interactions influence gel network density and mass transport properties.

#### 2.6.2. Protein Degradation by SEC-HPLC After Static In Vitro Digestion

**Figure 9:**
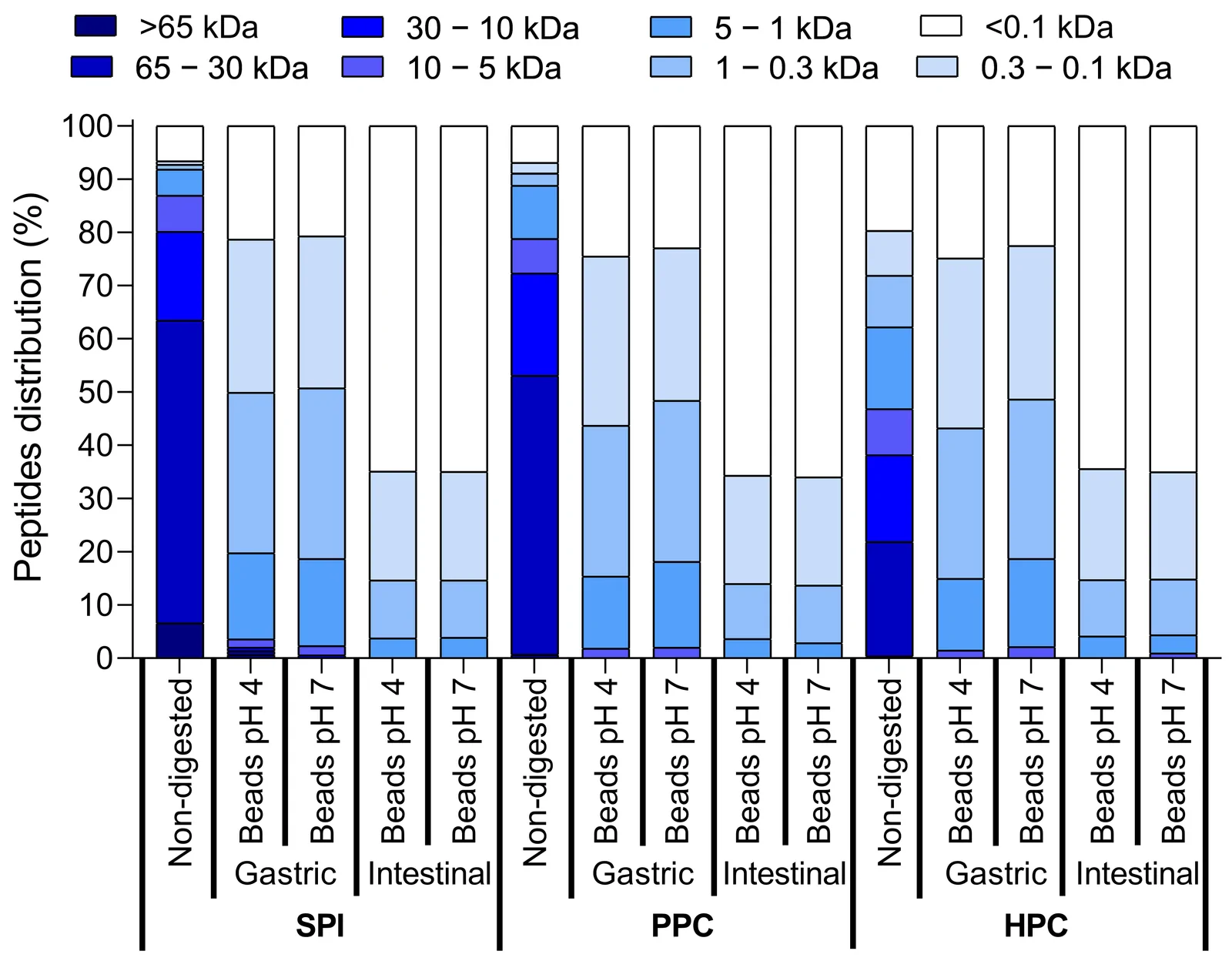
Relative molecular weight distribution of peptides (%) as determined by size exclusion chromatography, of non-digested soy protein isolate (SPI), pea protein concentrate (PPC) and hemp protein concentrate (HPC) and after in vitro gastric and gastro-intestinal digestion of alginate–protein beads produced at pH 4 and pH 7.

After the release of proteins from beads produced at two different pH and using different plant-based protein isolates, size exclusion chromatography was used to assess the relative peptide size distribution in both the undigested proteins and the released and solubilised in the bioaccessible fraction at the end of the gastric and intestinal digestion (Figure 9). The undigested protein isolates (soy (SPI), pea (PPC) and hemp (HPC)) showed a peptide size distribution characterised by high-molecular-weight fractions, primarily in the range of 10–65 kDa. This range likely includes albumins, which are typically found in soy, pea, and hemp proteins [70,71,72].

For the proteins released from beads produced at pH 4 and 7, substantial peptide breakdown by pepsin was observed during the gastric phase. Most peptides reduced to sizes below 5 kDa, with a predominant fraction further breaking down to sizes below 0.3 kDa, regardless of production pH or protein source.

During the gastric phase, a slight delay in peptide breakdown was evident in mid-sized ranges (1–0.3 kDa, 5–1 kDa, and 10–5 kDa). However, this delay seemed to be mitigated in the intestinal phase, where further degradation occurred due to the action of pancreatin proteases, mainly trypsin and chymotrypsin. The intestinal phase, particularly at pH 7, led to an increase in low-molecular-weight fractions (<0.1 kDa), suggesting extensive protein hydrolysis of the released proteins from the beads into the bioaccessible fractions. Interestingly, SPI exhibited a more pronounced reduction in peptide size across digestion phases, particularly for larger molecular weight ranges (>30 kDa), while HPC retained slightly higher values in the smaller molecular weight ranges post-digestion. This slight difference highlights variability in digestibility among protein sources, which may be leveraged in entrapment applications depending on the desired release profile and stability of the encapsulated bioactive compounds.

The HPLC-SEC chromatograms (Figure S1, Supplementary Material) show the molecular weight distribution of the fractions released during digestion. Consistent with the higher total protein release observed for the pH 4 formulations, the chromatographic profiles of SPI and PPC beads formed at pH 4 exhibited a higher proportion of lower-molecular-weight fragments compared to those formed at pH 7. This distribution indicates that the protein released from the pH 4 beads was hydrolysed into low-molecular-weight fractions and amino acids compared with the protein fractions released from the pH 7 beads. Unlike SPI and PPC, HPC showed highly similar chromatographic patterns at both gelation pH values, indicating that the initial gelation pH did not appreciably alter the peptide size distribution of the released hemp protein fractions during simulated digestion.

## 3. Conclusions

The results of this study show that the gelation pH modulates not only the strength but also the nature of the interactions between proteins and alginate, which in turn determines the retention and release behaviour of the proteins. Specifically, electrostatic attraction predominates in the case of HPC at pH 4, whilst hydrogen bonds, hydrophobic interactions and localised charge regions are more significant for SPI and PPC near their isoelectric points. These differences in interactions, together with pH-dependent changes in protein solubility, are associated with distinct final protein release profiles, such that beads formulated at pH 4 exhibit greater protein release during the intestinal phase than those prepared at pH 7. Overall, the gelation pH is a key processing parameter for modulating protein retention and final release in alginate-based delivery systems. These findings highlight the potential of SPI, PPC and HPC as functional components in alginate-based encapsulation systems. Future studies incorporating direct structural characterisation would further elucidate the microstructural mechanisms underlying the observed release behaviour.

## 4. Materials and Methods

### 4.1. Materials

Food-grade sodium alginate (Manugel^®^ GHB, batch GE305001; DuPont Nutrition & Biosciences ApS, Copenhagen, Denmark) was used in this study, pea protein concentrate (55%) obtained from IGV Foodtech (Nuthetal, Germany) and soy protein isolate (90%) from MyVegan (Manchester, UK), Hemp seed meal was kindly provided by Aceitera Dumont (Talagante, Chile). Pepsin was from porcine gastric mucosa (≥3200 units/mg protein, ref. P6887), pancreatin was from porcine pancreas (8 × USP specifications, ref. P7545) and bile was bovine (unfractionated, ref. B3883); the rest of chemicals used were acquired by Merck (Merck Sigma-Aldrich, St. Louis, MO, USA).

### 4.2. Hemp Protein Extraction

Hemp protein was isolated according to Piornos et al. [73] with modifications. The meal from *Cannabis sativa* L. seeds obtained from oil extraction residues was mixed with distilled water to obtain a 1:10 (*w*/*v*) solution. The sample was stirred at room temperature, and its pH was adjusted at 9.0 using 1 M NaOH. After 90 min, the suspension was centrifuged at 3200× *g* for 10 min using a Neofuge 15R centrifuge (Heal Force, Shanghai, China), the supernatant was recovered and readjusted at pH 4.3 with 1 M HCl. This solution was then centrifuged at 3200× *g* to separate the precipitated protein. Pellet was reconstituted in 1:5 (*w*/*w*) ratio using water and the pH was adjusted at 7.4. Finally, protein suspension was dried by spray drying in a B-290 mini-spray dryer (Büchi, Flawil, Switzerland). The spray dryer was set at 160 °C and 70 °C as the inlet and outlet temperatures, respectively, and a 5.3 g/min feed rate was applied. Protein content was determined using a LECO FP-628 analyser (LECO Corporation, St. Joseph, MI, USA) according to the Dumas method, resulting in a hemp protein concentrate (HPC) with 66% protein, nitrogen to protein conversion factor of 6.25 was used. After spray drying, the HPC powder was immediately transferred to airtight containers and stored at room temperature in desiccators until further analysis.

### 4.3. Purification of Pea Protein Concentrate

Purification was performed according to Comunian et al. [51]. Briefly, 10% (*w*/*w*) solution of pea protein (55%) was dispersed in distilled water and stirred for 3 h. The pH was adjusted to 8.0 with 1 M NaOH solution and kept overnight to ensure complete hydration. The sample was centrifuged at 10,000× *g* for 20 min at 25 °C using a Beckman Coulter Centrifuge, Avanti J-E (Beckman Coulter GmbH, Krefeld, Germany) and the supernatant filtered. After freezing, the pea protein solution was freeze-dried (Beta 1–8 LSCplus freeze-dryer/Martin Christ Gefriertrocknungsanlagen GmbH, Osterode am Harz, Germany). Following the drying step, a powder containing 69% protein was obtained, resulting in a pea protein concentrate (PPC). Protein content was quantified using the Dumas combustion technique with a Dumatherm N64+ analyser (Gerhardt, Königswinter, Germany). Nitrogen values were converted to protein using a factor of 6.25. After freeze-drying, the PPC powder was immediately placed in airtight containers and stored at room temperature in desiccators until further analysis.

### 4.4. Protein Solubility

The protein solubility assay was carried out according to Du et al. [74] with modifications. HPC, PPC, and SPI (10 g/L) were dissolved in distilled water and stirred for 2 h at room temperature. The solutions were adjusted to pH values from 2 to 7, in increments of 1 unit using 1 M HCl or NaOH. The samples were centrifuged at 10,000× *g* for 20 min at room temperature to remove insoluble material. Supernatant was recovered and diluted in distilled water 1:10 (*v*/*v*). The protein quantification was carried out using a commercial protein kit (PierceTM BCA Protein Assay Kit) as follows: 25 µL of the supernatant diluted in 200 µL of the BCA working reagent was mixed in a 96-well microplate, which was covered and incubated for 30 min at 37 °C in a thermoshaker. A standard curve was created using Bovine Serum Albumin (BSA) over a concentration range of 0–2 g/L. The absorbance was measured at 562 nm using a spectrophotometer (Molecular Devices, SpectraMax ABS Plus, USA). Protein solubility was calculated as follows in Equation (1).

(1) $$Protein\;solubility\;(\%)=\left(\frac{C_{s}}{C_{t}}\right)\times 100\%$$

where *C_s_* is the protein concentration in the supernatant (soluble fraction), determined using the BCA assay, and *C_t_* is the total protein concentration originally added to the solution.

### 4.5. Zeta Potential

The apparent surface charge (zeta potential) measurements of HPC, SPI and PPC solutions were performed as reported by Tang et al. [36]. Solutions of sodium alginate, HPC SPI and PPC were prepared at 0.1% *w*/*v* at different pH values, from pH 2 to 7 in increments of 1 unit using 1 M HCl or NaOH. After centrifugation at 8000× *g* for 20 min at room temperature, the supernatant was carefully collected, and its zeta potential was determined using a Zetasizer Nano ZS (Malvern Instruments Ltd., Worcestershire, UK).

### 4.6. Fourier Transform Infrared (FTIR) Spectroscopy

FTIR analysis of the samples was performed using an ATR-FTIR Spectrum One spectrometer (PerkinElmer Inc., Waltham, MA, USA). Solutions of HPC, PPC, SPI, and alginate were prepared at a concentration of 0.1% (*w*/*v*) in distilled water. Each protein solution was then mixed with the alginate solution in a 1:1 (*v*/*v*) ratio. Prior to measurement, each protein–alginate mixture was freeze-dried for 48 h. The resulting dry powder was placed directly onto the ATR diamond crystal and clamped under pressure at room temperature. Spectra were acquired over the range of 4000–600 cm^−1^ with a resolution of 4 cm^−1^ and 30 scans per sample. All measurements were performed in triplicate. To identify specific molecular interactions between proteins and alginate, difference spectra were generated following the spectral subtraction approach described by Kehoe et al. [58]. Briefly, spectra of the individual proteins, alginate, and the corresponding protein–alginate mixtures were baseline-corrected and vector-normalized using SpectraGryph 1.2 software (Friedrich Menges Software, Oberstdorf, Germany). Difference spectra were obtained by subtracting the weighted sum of the normalized protein and alginate spectra from the normalized spectrum of the corresponding protein–alginate mixture. Since protein and alginate solutions were prepared at the same concentration (0.1%, *w*/*v*) and mixed at a 1:1 (*v*/*v*) ratio prior to freeze-drying, equal weighting factors (0.5 for protein and 0.5 for alginate) were applied during spectral subtraction. Difference spectra were calculated independently for each freeze-dried replicate.

### 4.7. Bead Production

A 2% (*w*/*v*) aqueous solution of sodium alginate was prepared by slowly dissolving the powder in distilled water at room temperature during a 2 h period. SPI, HPC, and PPC (2% *w*/*v*) and alginate (2% *w*/*v*) were mixed in 1:1 (*v*/*v*) ratio with continuous stirring to form a uniform solution for 1 h. The mixed solutions of alginate and PPC, HPC, or SPI were adjusted at pH 4 and 7 prior to bead formation with 1 M HCl or NaOH. The solutions were dripped into a 250 mL glass bath with 250 mM CaCl_2_ using a syringe with needle (21G × 38 mm). The solution was gently mixed using a magnetic stirrer at 330 rpm. Crosslinking was carried out for 1 h at room temperature with continuous stirring. The resulting hydrogel beads were recovered by filtration and washed with distilled water to remove residual Ca^2+^ adhering to their surfaces.

### 4.8. Entrapment Efficiency (EE)

To determine the protein entrapment efficiency, the methodology by Machado et al. [10] was used. Alginate–protein beads were suspended and stirred at 100 rpm in 3% (*w*/*v*) sodium citrate until complete dissolution was achieved for 30 min at 37 °C. The BCA protein assay was used to determine the protein content of the beads. *EE* (%) was calculated using Equation (2):

(2) $$EE\;\left(\%\right)=\frac{mass\;of\;protein\;encapsulated}{mass\;of\;load\;protein\;protein/alginate\;stock\;solution}\;\times 100\%$$

### 4.9. Structural Analysis

Confocal laser scanning microscopy (CLSM) was used to observe the microstructure of the hydrogel beads. Samples were stained using 0.1% Fluorescein isothiocyanate (FITC) and 0.1% Fast Green for alginate and protein, respectively. The beads were left overnight at 5 °C for complete diffusion of the stain through the structure. The stained samples were observed using a Leica TCS SP5 confocal microscope (Leica Microsystems GmbH, Wetzlar, Germany). Excitation was provided by an argon laser (488 nm), whereas fluorescence emission was detected using a HeNe laser (633 nm).

The diameter of the beads (*d*) was measured as follows: photographs were taken using a camera (iPhone 12 Pro Max, Apple Inc., California, USA). Samples were placed in a 9 cm plastic Petri dish, and images were analysed using ImageJ software (v1.53, Bethesda, MD, USA). The projected area (A) of each bead was determined, and the diameter of a circle with an equivalent projected area was calculated according to Equation (3):

(3) *d = 2 × (A/π)*^1/2^

where *A* is the projected area (mm^2^) of the bead. Subsequently, the Sphericity Factor (*SF*) was calculated according to Equation (4):

(4) *SF = 4πA/P*^2^

where *A* is the projected area (mm^2^) and *P* is the perimeter (mm) of the bead. An *SF* value equal to 1 indicates a perfect spherical shape.

### 4.10. Static In Vitro Digestion

Enzyme activities were assayed according to Brodkorb et al. [75] and Minekus et al. [76]. All samples were digested in vitro using the static INFOGEST protocol in independent tubes.

Oral phase: 2 g of beads were mixed with 1.6 mL of simulated salivary fluid (SSF), 10 µL of CaCl_2_ (300 mM), and 390 µL of water to reach a final volume of 4 mL. The mixture was incubated at 37 °C for 2 min.

Gastric phase: 3.2 mL of simulated gastric fluid (SGF), 200 µL of pepsin solution, and 2 µL of CaCl_2_ (300 mM) were added to the oral phase product. Then, 1 M HCl was added to adjust the mixture to pH 3, and the exact volume required for this adjustment was recorded. Finally, the volume was brought to 8 mL, and the samples were incubated at 37 °C for 120 min.

Intestinal phase: the gastric chyme was mixed with 3.4 mL of simulated intestinal fluid (SIF), 2 mL of pancreatin solution, 1 mL of bile solution, and 16 µL of CaCl_2_ (300 mM). The pH was adjusted to 7 using 1 M NaOH, and the exact volume required for this adjustment was recorded. The mixture was made up to a final volume of 16 mL with distilled water and incubated at 37 °C for 120 min.

Every in vitro digestion phase was performed under constant gentle mixing (15 rpm) on a rotating wheel (Stuart rotator SB3, Stuart Equipment, Cole-Parmer, UK). A protein-free blank (water) was digested in parallel. Enzyme reaction at the end of every phase (gastric and intestinal) were stopped by heat-treatment (100 °C for 5 min). The experiments were performed in independent triplicate and aliquots kept frozen at −20 °C for further analysis.

### 4.11. Protein Release

Protein released after the gastric and intestinal phases was quantified in the corresponding supernatants using a commercial protein assay kit (Pierce™ BCA Protein Assay Kit). Prior to analysis, supernatants were diluted 1× and 10× with distilled water for the gastric and intestinal phases, respectively. Aliquots (25 µL) of the diluted supernatants were mixed with 200 µL of BCA working reagent in a 96-well microplate, covered, and incubated for 30 min at 37 °C in a thermoshaker. Absorbance was measured at 562 nm using a spectrophotometer (SpectraMax ABS Plus, Molecular Devices, USA). Protein release was expressed as a percentage and calculated according to Equation (5).

(5) $$Protein\;release\;\left(\%\right)=\left(\frac{m_{release}}{m_{entrapped}}\right)\times 100\%$$

where $m_{release}$ is the mass of protein quantified in the supernatant by the BCA assay after correction for the background protein signal determined from a digestion blank containing all simulated digestive fluids and enzymes, but no HPC, PPC or SPI, processed in parallel. The denominator, $m_{entrapped}$, is the mass of protein entrapped within the beads prior to digestion, calculated from the initial theoretical protein load and the entrapment efficiency (EE, Section 4.8).

### 4.12. High-Performance Size Exclusion Chromatography (SEC)

For the peptide size distribution of the bioaccessible fraction, size exclusion chromatography (SEC) was carried out. An HPLC system (1260 Infinity II, Agilent Technologies, Santa Clara, CA, USA), a multiple wavelength detector (MWD) and a TSKgel G2000SWxl column (600 mm × 7.8 mm, 5 µm, 125 A, Tosoh Bioscience, Tokyo, Japan) were used. Before SEC analysis, protein dispersions were adjusted to 0.25% (*w*/*v*) and clarified by filtration through 0.22 µm PES membranes. Separation was performed under isocratic elution with a mobile phase of 30% acetonitrile containing 0.1% trifluoroacetic acid at a constant flow rate of 0.5 mL/min, with UV detection at 214 nm. Apparent peptide molecular weights were assigned by comparing retention times with a calibration set consisting of bovine serum albumin (67 kDa), carbonic anhydrase (29 kDa), β-lactoglobulin (18.4 kDa), aprotinin (6.5 kDa), His–Leu (0.26 kDa), and glycine (0.075 kDa).

### 4.13. Statistical Analysis

Measurements were carried out in triplicate, and the results are reported as mean ± standard error. Bead size and shape were determined from measurements of more than 50 beads for each sample. Statistical significance among treatments was evaluated using Tukey’s post hoc test with a significance threshold of *p* < 0.05. Data processing was performed using Minitab 21.4 (Minitab Inc., State College, PA, USA).

## Data Availability

The raw data supporting the conclusions of this article will be made available by the authors on request.

## Supplementary Materials

The following supporting information can be downloaded at: https://www.mdpi.com/article/10.3390/gels12080717/s1, Figure S1: HPLC-SEC chromatograms of non-digested SPI (A), PPC (B), and HPC (C), and of the fractions released after the gastric (Gast) and intestinal (Int) phases of in vitro digestion of alginate–protein beads prepared at pH 4 and pH 7.

## References

[1] Batista P.S.P., De Morais A.M.M.B., Pintado M.M.E., De Morais R.M.S.C., Cohen E., Merzendorfer H. (2019). Alginate: Pharmaceutical and Medical Applications. Extracellular Sugar-Based Biopolymers Matrices.

[2] Bi D., Yang X., Yao L., Hu Z., Li H., Xu X., Lu J. (2022). Potential Food and Nutraceutical Applications of Alginate: A Review. Mar. Drugs.

[3] Draget K.I., Østgaard K., Smidsrød O. (1990). Homogeneous Alginate Gels: A Technical Approach. Carbohydr. Polym..

[4] Farshidfar N., Iravani S., Varma R.S. (2023). Alginate-Based Biomaterials in Tissue Engineering and Regenerative Medicine. Mar. Drugs.

[5] Draget K.I., Skjåk Bræk G., Smidsrød O. (1994). Alginic Acid Gels: The Effect of Alginate Chemical Composition and Molecular Weight. Carbohydr. Polym..

[6] Neiser S., Draget K.I., Smidsrød O. (1999). Interactions in Bovine Serum Albumin–Calcium Alginate Gel Systems. Food Hydrocoll..

[7] Grant G.T., Mon E.R., Rees S.D.A. (1973). Biological Interactions between Polysaccharides and Divalent Cations: The Egg-Box Model. FEBS Lett..

[8] Madsen M., Rønne M.E., Li R., Greco I., Ipsen R., Svensson B. (2022). Simulated Gastrointestinal Digestion of Protein Alginate Complexes: Effects of Whey Protein Cross-Linking and the Composition and Degradation of Alginate. Food Funct..

[9] Zhu Y., Li J., Liu S., Yang H., Lu F., Zhu M. (2025). Quinoa Protein/Sodium Alginate Complex-Stabilized Pickering Emulsion for Sustained Release of Curcumin and Enhanced Anticancer Activity Against HeLa Cells. Foods.

[10] Machado A.R., Silva P.M.P., Vicente A.A., Souza-Soares L.A., Pinheiro A.C., Cerqueira M.A. (2022). Alginate Particles for Encapsulation of Phenolic Extract from Spirulina Sp. LEB-18: Physicochemical Characterization and Assessment of In Vitro Gastrointestinal Behavior. Polymers.

[11] Rezagholizade-shirvan A., Soltani M., Shokri S., Radfar R., Arab M., Shamloo E. (2024). Bioactive Compound Encapsulation: Characteristics, Applications in Food Systems, and Implications for Human Health. Food Chem. X.

[12] Ganguly S., Mondal S., Das P., Bhawal P., Maity P.P., Ghosh S., Dhara S., Das N.C. (2018). Design of Psyllium-g-Poly(Acrylic Acid-Co-Sodium Acrylate)/Cloisite 10A Semi-IPN Nanocomposite Hydrogel and Its Mechanical, Rheological and Controlled Drug Release Behaviour. Int. J. Biol. Macromol..

[13] Xu Y., Yan X., Zheng H., Li J., Wu X., Xu J., Zhen Z., Du C. (2024). The Application of Encapsulation Technology in the Food Industry: Classifications, Recent Advances, and Perspectives. Food Chem. X.

[14] Sieders M., Candry P., El Aidy S. (2025). Hydrogel-Based Experimental Models of the Gastrointestinal Tract. Microbiome.

[15] Kolotova D.S., Borovinskaya E.V., Bordiyan V.V., Zuev Y.F., Salnikov V.V., Zueva O.S., Derkach S.R. (2023). Phase Behavior of Aqueous Mixtures of Sodium Alginate with Fish Gelatin: Effects of pH and Ionic Strength. Polymers.

[16] Pedrali D., Scarafoni A., Giorgi A., Lavelli V. (2023). Binary Alginate-Whey Protein Hydrogels for Antioxidant Encapsulation. Antioxidants.

[17] Zare M., Golmakani M.-T., Niakousari M., Eskandari M.H., Ghiasi F., Hosseini S.M.H. (2024). Alginate/Whey Protein Isolate-Based Emulgel as an Alternative Margarine Replacer in Processed Cheese: Impact on Rheological, Mechanical, Nutritional, and Sensory Characteristics. J. Dairy Sci..

[18] Zhang Z., Zhang R., Zou L., McClements D.J. (2016). Protein Encapsulation in Alginate Hydrogel Beads: Effect of pH on Microgel Stability, Protein Retention and Protein Release. Food Hydrocoll..

[19] Asledottir T., Vegarud G.E., Picariello G., Mamone G., Lea T.E., Røseth A., Ferranti P., Devold T.G. (2023). Bioactive Peptides Identified in Pea and Faba Bean after in Vitro Digestion with Human Gastrointestinal Enzymes. J. Funct. Foods.

[20] Baugreet S., Gomez C., Auty M.A.E., Kerry J.P., Hamill R.M., Brodkorb A. (2019). In Vitro Digestion of Protein-Enriched Restructured Beef Steaks with Pea Protein Isolate, Rice Protein and Lentil Flour Following Sous Vide Processing. Innov. Food Sci. Emerg. Technol..

[21] Hadidi M., Aghababaei F., Gonzalez-Serrano D.J., Goksen G., Trif M., McClements D.J., Moreno A. (2024). Plant-Based Proteins from Agro-Industrial Waste and by-Products: Towards a More Circular Economy. Int. J. Biol. Macromol..

[22] Sarathy S.P., Ravikumar H., Nanjan P., Alagesan N., Chua B.L. (2025). Plant-Based Protein: A Multi-Nutritional Sustainable Alternative to Animal Foods and Their Structure, Functions, and Relationship: A Review. Int. J. Biol. Macromol..

[23] Xu Y., Sismour E., Britland J.W., Sellers A., Abraha-Eyob Z., Yousuf A., Rao Q., Kim J., Zhao W. (2022). Physicochemical, Structural, and Functional Properties of Hemp Protein vs Several Commercially Available Plant and Animal Proteins: A Comparative Study. ACS Food Sci. Technol..

[24] Xiang L., Jiang B., Xiong Y.L., Zhou L., Zhong F., Zhang R., Bin Tahir A., Xiao Z. (2023). Beany Flavor in Pea Protein: Recent Advances in Formation Mechanism, Analytical Techniques and Microbial Fermentation Mitigation Strategies. Food Biosci..

[25] Karabulut G., Kahraman O., Pandalaneni K., Kapoor R., Feng H. (2023). A Comprehensive Review on Hempseed Protein: Production, Functional and Nutritional Properties, Novel Modification Methods, Applications, and Limitations. Int. J. Biol. Macromol..

[26] Capcanari T., Covaliov E., Negoița C. (2025). Harnessing Hemp (*Cannabis sativa* L.) Seed Cake Proteins: From Concentrate Production to Enhanced Choux Pastry Quality. Foods.

[27] Capcanari T., Covaliov E., Negoița C., Siminiuc R., Chirsanova A., Reșitca V., Țurcanu D. (2023). Hemp Seed Cake Flour as a Source of Proteins, Minerals and Polyphenols and Its Impact on the Nutritional, Sensorial and Technological Quality of Bread. Foods.

[28] Zhang Z., Zhang R., McClements D.J. (2017). Control of Protein Digestion under Simulated Gastrointestinal Conditions Using Biopolymer Microgels. Food Res. Int..

[29] Christensen H.N. (1966). Proteins as buffers. Ann. N. Y. Acad. Sci..

[30] Mennah-Govela Y.A., Singh R.P., Bornhorst G.M. (2019). Buffering Capacity of Protein-Based Model Food Systems in the Context of Gastric Digestion. Food Funct..

[31] Eichhorn M., Thorenz E., Drusch S. (2025). Behaviour of Potato Protein-Pectin Conjugates in Emulsions: Insights from Interfacial Shear Rheology. Food Hydrocoll..

[32] Kramer R.M., Shende V.R., Motl N., Pace C.N., Scholtz J.M. (2012). Toward a Molecular Understanding of Protein Solubility: Increased Negative Surface Charge Correlates with Increased Solubility. Biophys. J..

[33] Trevino S.R., Scholtz J.M., Pace C.N. (2008). Measuring and Increasing Protein Solubility. J. Pharm. Sci..

[34] Zayas J.F. (1997). Solubility of Proteins. Functionality of Proteins in Food.

[35] Dumetz A.C., Chockla A.M., Kaler E.W., Lenhoff A.M. (2008). Effects of pH on Protein–Protein Interactions and Implications for Protein Phase Behavior. Biochim. Biophys. Acta (BBA) Proteins Proteom..

[36] Tang Q., Roos Y.H., Miao S. (2023). Plant Protein versus Dairy Proteins: A pH-Dependency Investigation on Their Structure and Functional Properties. Foods.

[37] Kepekçi R.A., İlçe B.Y., Kanmazalp S.D., Atta-ur-Rahman (2021). Chapter 8-Plant-Derived Biomaterials for Wound Healing. Bioactive Natural Products.

[38] Marsalek R. (2014). Particle Size and Zeta Potential of ZnO. APCBEE Procedia.

[39] Pate K., Safier P. (2016). Chemical Metrology Methods for CMP Quality. Advances in Chemical Mechanical Planarization (CMP).

[40] Bokkhim H., Bansal N., Grøndahl L., Bhandari B. (2015). Interactions between Different Forms of Bovine Lactoferrin and Sodium Alginate Affect the Properties of Their Mixtures. Food Hydrocoll..

[41] Choukaife H., Doolaanea A.A., Alfatama M. (2020). Alginate Nanoformulation: Influence of Process and Selected Variables. Pharmaceuticals.

[42] Longo G.S., Pérez-Chávez N.A., Szleifer I. (2019). How Protonation Modulates the Interaction between Proteins and pH-Responsive Hydrogel Films. Curr. Opin. Colloid Interface Sci..

[43] Qing R., Hao S., Smorodina E., Jin D., Zalevsky A., Zhang S. (2022). Protein Design: From the Aspect of Water Solubility and Stability. Chem. Rev..

[44] Adrar N., Ceylan F.D., Capanoglu E. (2024). Hazelnut Protein and Sodium Alginate Complex Coacervates: An Effective Tool for the Encapsulation of the Hydrophobic Polyphenol Quercetin. ACS Omega.

[45] Barth A. (2007). Infrared Spectroscopy of Proteins. Biochim. Biophys. Acta (BBA) Bioenerg..

[46] Jackson M., Mantsch H.H. (1995). The Use and Misuse of FTIR Spectroscopy in the Determination of Protein Structure. Crit. Rev. Biochem. Mol. Biol..

[47] Lefèvre T., Subirade M. (2000). Molecular Differences in the Formation and Structure of Fine-Stranded and Particulate β-Lactoglobulin Gels. Biopolymers.

[48] Zhao Y., Li F., Carvajal M.T., Harris M.T. (2009). Interactions between Bovine Serum Albumin and Alginate: An Evaluation of Alginate as Protein Carrier. J. Colloid Interface Sci..

[49] Ji Y., Yang X., Ji Z., Zhu L., Ma N., Chen D., Jia X., Tang J., Cao Y. (2020). DFT-Calculated IR Spectrum Amide I, II, and III Band Contributions of N-Methylacetamide Fine Components. ACS Omega.

[50] Cortez-Trejo M.C., Figueroa-Cárdenas J.D., Quintanar-Guerrero D., Baigts-Allende D.K., Manríquez J., Mendoza S. (2022). Effect of pH and Protein-Polysaccharide Ratio on the Intermolecular Interactions between Amaranth Proteins and Xanthan Gum to Produce Electrostatic Hydrogels. Food Hydrocoll..

[51] Comunian T.A., Archut A., Gomez-Mascaraque L.G., Brodkorb A., Drusch S. (2022). The Type of Gum Arabic Affects Interactions with Soluble Pea Protein in Complex Coacervation. Carbohydr. Polym..

[52] Carpenter J.F., Crowe J.H. (1989). An Infrared Spectroscopic Study of the Interactions of Carbohydrates with Dried Proteins. Biochemistry.

[53] Ding Z., Jiang F., Liu K., Gong F., Liu Y., Zheng Z., Xu Y.-J. (2023). Structural and Functional Characteristics of Hemp Protein Isolate–Pullulan Polysaccharide Glycosylation Conjugate in an Aqueous Model System. Foods.

[54] Lee K.Y., Mooney D.J. (2012). Alginate: Properties and Biomedical Applications. Prog. Polym. Sci..

[55] Dickinson E. (1998). Stability and Rheological Implications of Electrostatic Milk Protein–Polysaccharide Interactions. Trends Food Sci. Technol..

[56] Turgeon S.L., Schmitt C., Sanchez C. (2007). Protein–Polysaccharide Complexes and Coacervates. Curr. Opin. Colloid Interface Sci..

[57] Guerrero P., Kerry J.P., De La Caba K. (2014). FTIR Characterization of Protein–Polysaccharide Interactions in Extruded Blends. Carbohydr. Polym..

[58] Kehoe J.J., Remondetto G.E., Subirade M., Morris E.R., Brodkorb A. (2008). Tryptophan-Mediated Denaturation of β-Lactoglobulin A by UV Irradiation. J. Agric. Food Chem..

[59] Chan E.-S., Lee B.-B., Ravindra P., Poncelet D. (2009). Prediction Models for Shape and Size of Ca-Alginate Macrobeads Produced through Extrusion–Dripping Method. J. Colloid Interface Sci..

[60] Lee B.-B., Ravindra P., Chan E.-S. (2013). Size and Shape of Calcium Alginate Beads Produced by Extrusion Dripping. Chem. Eng. Technol..

[61] Chuang J.-J., Huang Y.-Y., Lo S.-H., Hsu T.-F., Huang W.-Y., Huang S.-L., Lin Y.-S. (2017). Effects of pH on the Shape of Alginate Particles and Its Release Behavior. Int. J. Polym. Sci..

[62] Hua S., Ma H., Li X., Yang H., Wang A. (2010). PH-Sensitive Sodium Alginate/Poly(Vinyl Alcohol) Hydrogel Beads Prepared by Combined Ca^2+^ Crosslinking and Freeze-Thawing Cycles for Controlled Release of Diclofenac Sodium. Int. J. Biol. Macromol..

[63] Liu X., Xue F., Adhikari B. (2024). Recent Advances in Plant Protein Modification: Spotlight on Hemp Protein. Sustain. Food Technol..

[64] Eckhardt L., Bu F., Franczyk A., Michaels T., Ismail B.P. (2024). Hemp (*Cannabis sativa* L.) Protein: Impact of Extraction Method and Cultivar on Structure, Function, and Nutritional Quality. Curr. Res. Food Sci..

[65] Gentile L. (2020). Protein–Polysaccharide Interactions and Aggregates in Food Formulations. Curr. Opin. Colloid Interface Sci..

[66] Davis P.D., Parbrook G.D., Kenny G.N.C. (1995). Diffusion and Osmosis. Basic Physics and Measurement in Anaesthesia.

[67] Anal A.K., Bhopatkar D., Tokura S., Tamura H., Stevens W.F. (2003). Chitosan-Alginate Multilayer Beads for Gastric Passage and Controlled Intestinal Release of Protein. Drug Dev. Ind. Pharm..

[68] Massana Roquero D., Bollella P., Katz E., Melman A. (2021). Controlling Porosity of Calcium Alginate Hydrogels by Interpenetrating Polyvinyl Alcohol–Diboronate Polymer Network. ACS Appl. Polym. Mater..

[69] Martinsen A., Storrø I., Skjårk-Bræk G. (1992). Alginate as Immobilization Material: III. Diffusional Properties. Biotech. Bioeng..

[70] Dziuba J., Szerszunowicz I., Nałęcz D., Dziuba M. (2014). Proteomic Analysis of Albumin and Globulin Fractions of Pea (*Pisum sativum* L.) Seeds. Acta Sci. Pol. Technol. Aliment.

[71] Malomo S.A., Aluko R.E. (2015). A Comparative Study of the Structural and Functional Properties of Isolated Hemp Seed (*Cannabis sativa* L.) Albumin and Globulin Fractions. Food Hydrocoll..

[72] Singh A., Meena M., Kumar D., Dubey A.K., Hassan M.I. (2015). Structural and Functional Analysis of Various Globulin Proteins from Soy Seed. Crit. Rev. Food Sci. Nutr..

[73] Piornos J.A., Burgos-Díaz C., Ogura T., Morales E., Rubilar M., Maureira-Butler I., Salvo-Garrido H. (2015). Functional and Physicochemical Properties of a Protein Isolate from Alu Prot -CGNA: A Novel Protein-Rich Lupin Variety (*Lupinus luteus*). Food Res. Int..

[74] Du H., Lin Y., Stanton C., Daniloski D., Zannini E., Ross R.P., Miao S. (2023). Characterization and Functional Properties of pH- and Heated Time-Induced Aggregates from Red Lentil Protein. Food Struct..

[75] Brodkorb A., Egger L., Alminger M., Alvito P., Assunção R., Ballance S., Bohn T., Bourlieu-Lacanal C., Boutrou R., Carrière F. (2019). INFOGEST Static in Vitro Simulation of Gastrointestinal Food Digestion. Nat. Protoc..

[76] Minekus M., Alminger M., Alvito P., Ballance S., Bohn T., Bourlieu C., Carrière F., Boutrou R., Corredig M., Dupont D. (2014). A Standardised Static *in vitro* Digestion Method Suitable for Food—An International Consensus. Food Funct..

