# Supplementary material for: Effect of Gelation pH on the Entrapment and In Vitro Gastrointestinal Digestion of Plant Proteins in Alginate Beads
Source: Gels. 2026 Aug 13;12(8):717. doi: 10.3390/gels12080717 (PMC13512790; doi:10.3390/gels12080717)
Supplement: Supplementary file 1 [file gels-12-00717-s001.zip › gels-4465921-supplementary.pdf]

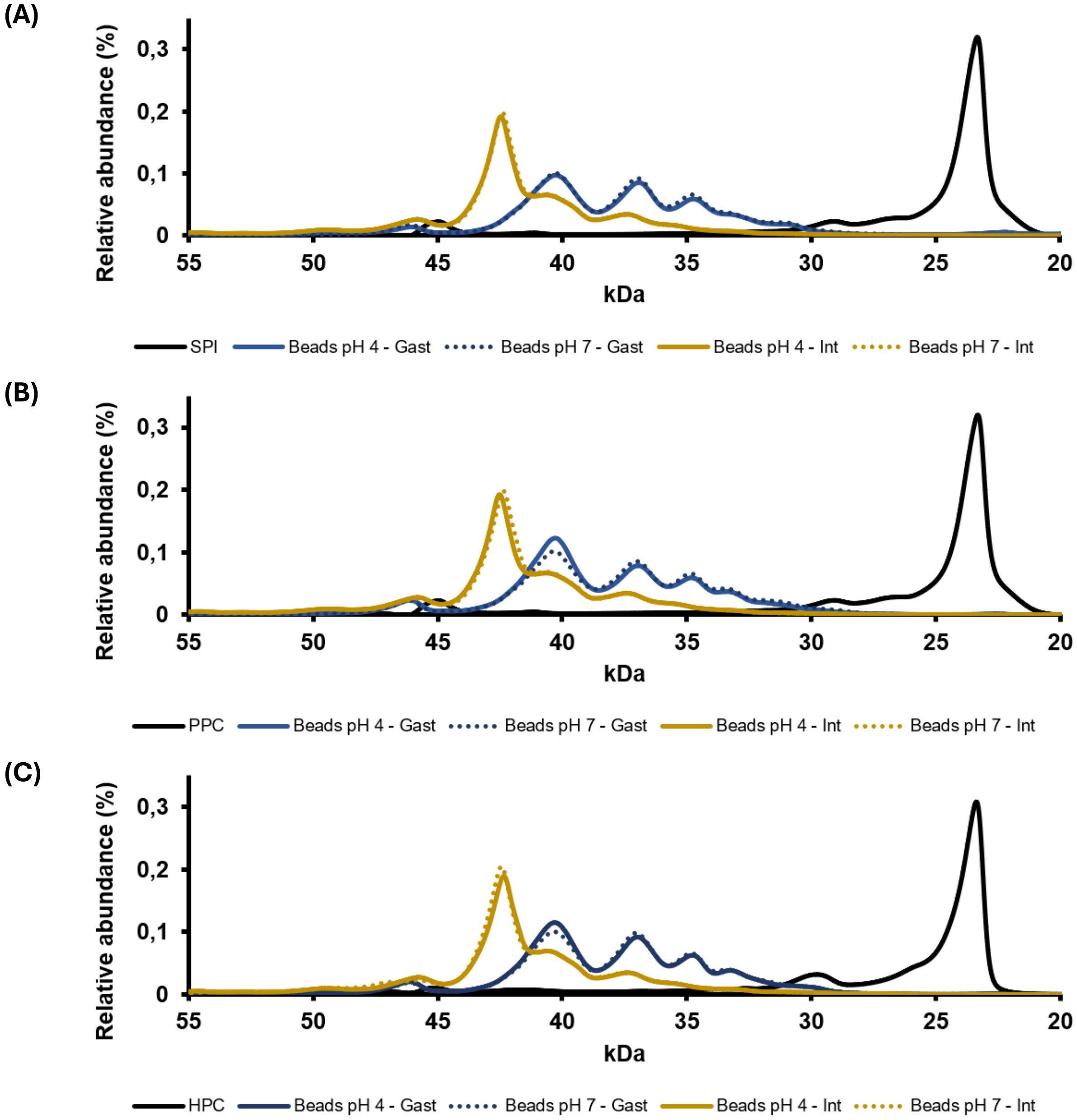

Figure S1. HPLC-SEC chromatograms of non-digested SPI (A), PPC (B), and HPC (C), and of the fractions released after the gastric (Gast) and intestinal (Int) phases of *in vitro* digestion of alginate–protein beads prepared at pH 4 and pH 7.
